# Long-Term Outcome After Out-of-Hospital Cardiac Arrest: An Utstein-Based Analysis

**DOI:** 10.3389/fcvm.2021.764043

**Published:** 2021-12-15

**Authors:** Enrico Baldi, Sara Compagnoni, Stefano Buratti, Roberto Primi, Sara Bendotti, Alessia Currao, Francesca Romana Gentile, Giuseppe Maria Sechi, Claudio Mare, Roberta Bertona, Irene Raimondi Cominesi, Erika Taravelli, Cristian Fava, Gian Battista Danzi, Luigi Oltrona Visconti, Simone Savastano, all the Lombardia CARe Researchers, Guido Francesco Villa

**Affiliations:** ^1^Section of Cardiology, Department of Molecular Medicine, University of Pavia, Pavia, Italy; ^2^Cardiac Intensive Care Unit, Arrhythmia and Electrophysiology and Experimental Cardiology, Fondazione Istituto di Ricovero e Cura a Carattere Scientifico (IRCCS) Policlinico San Matteo, Pavia, Italy; ^3^Division of Cardiology, Fondazione Istituto di Ricovero e Cura a Carattere Scientifico (IRCCS) Policlinico San Matteo, Pavia, Italy; ^4^Division of Cardiology, Ospedale Civile di Voghera, Azienda Socio-Sanitaria Territoriale (ASST) di Pavia, Voghera, Italy; ^5^Agenzia Regionale Emergenza Urgenza, Milan, Italy; ^6^Division of Cardiology, Ospedale Civile di Vigevano, Azienda Socio-Sanitaria Territoriale (ASST) di Pavia, Vigevano, Italy; ^7^Division of Cardiology, Ospedale Maggiore di Lodi, Azienda Socio-Sanitaria Territoriale (ASST) di Lodi, Lodi, Italy; ^8^Division of Cardiology, Ospedale Maggiore di Crema, Azienda Socio-Sanitaria Territoriale (ASST) di Crema, Crema, Italy; ^9^Division of Cardiology, Ospedale Carlo Poma, Azienda Socio-Sanitaria Territoriale (ASST) di Mantova, Mantova, Italy; ^10^Division of Cardiology, Ospedale di Cremona, Azienda Socio-Sanitaria Territoriale (ASST) di Cremona, Cremona, Italy

**Keywords:** out of hospital cardiac arrest, survivors, long-term outcomes, mortality, Utstein

## Abstract

**Background:** No data are available regarding long-term survival of out-of-hospital cardiac arrest (OHCA) patients based on different Utstein subgroups, which are expected to significantly differ in terms of survival. We aimed to provide the first long-term survival analysis of OHCA patients divided according to Utstein categories.

**Methods:** We analyzed all the 4,924 OHCA cases prospectively enrolled in the Lombardia Cardiac Arrest Registry (Lombardia CARe) from 2015 to 2019. Pre-hospital data, survival, and cerebral performance category score (CPC) at 1, 6, and 12 months and then every year up to 5 years after the event were analyzed for each patient.

**Results:** A decrease in survival was observed during the follow-up in all the Utstein categories. The risk of death of the “all-EMS treated” group exceeded the general population for all the years of follow-up with standardized mortality ratios (SMRs) of 23 (95%CI, 16.8–30.2), 6.8 (95%CI, 3.8–10.7), 3.8 (95%CI, 1.7–6.7), 4.05 (95%CI, 1.9–6.9), and 2.6 (95%CI, 1.03–4.8) from the first to the fifth year of follow-up. The risk of death was higher also for the Utstein categories “shockable bystander witnessed” and “shockable bystander CPR”: SMRs of 19.4 (95%CI, 11.3–29.8) and 19.4 (95%CI, 10.8–30.6) for the first year and of 6.8 (95%CI, 6.6–13) and 8.1 (95%CI, 3.1–15.3) for the second one, respectively. Similar results were observed considering the patients discharged with a CPC of 1–2.

**Conclusions:** The mortality of OHCA patients discharged alive from the hospital is higher than the Italian standard population, also considering those with the most favorable OHCA characteristics and those discharged with good neurological outcome. Long-term follow-up should be included in the next Utstein-style revision.

## Introduction

Out-of-hospital cardiac arrest (OHCA) is the leading cause of death at least in high-income countries (1). Survival is affected by early cardio-pulmonary resuscitation (CPR) and defibrillation together with a good strategy of in-hospital care, that constitute the cardiac arrest rescue system, nicely summarized by the chain of survival (2). Patients' outcome after OHCA differs considerably by regions (3, 4), mainly as a result of system performance improvements (5, 6). High-quality OHCA registries with a uniform collecting system are crucial to compare epidemiology, effect of treatments, and outcome in different regions aiming to monitor performance and improve survival. For these reasons, the International Liaison Committee on Resuscitation (ILCOR) proposed the Utstein template in 1990 (7), a complex of general rules for collecting and exposing OHCA data, which was then updated in 2004 (8), 2007 (9), and 2014 (10). Based on these recommendations many Utstein-based out-of-hospital cardiac arrest registries were established worldwide in the last 10 years but, unfortunately, with a short follow-up of only 1 month at least for the majority of them. Albeit the ILCOR 2015 Consensus Statement (10) considers the survival at 12 months and beyond supplemental information, a long-term follow-up could provide useful insights about long-term issues of survivors improving their treatment. The Lombardia Cardiac Arrest Registry (Lombardia CARe), a prospective cardiac arrest registry in northern Italy, accepted the challenge of providing long-term follow-up in 2015, by planning a follow-up of 5 years after the event (11). Only a few datasets are available regarding such a long-term survival of OHCA patients and they concern mainly patients with specific features: hypertrophic cardiomyopathy (12), STEMI (13, 14), OHCA patients receiving early defibrillation (15), and patients with idiopathic ventricular fibrillation (IVF) (16). However, no data are available so far about patients' long-term outcome in the different Utstein subgroups, which are expected to significantly differ in terms of survival. Our aim was to provide the first analysis about long-term survival of OHCA patients divided according Utstein categories.

## Materials and Methods

We considered for analysis all the OHCA cases prospectively enrolled in Lombardia CARe from January 1, 2015 to December 31, 2019. We evaluated for each patient all the data regarding the pre-hospital treatment, the survival, and the cerebral performance category score (CPC) at 1 month, 6 months, 12 months, and then every year up to 5 years after the event.

### Lombardia CARe

The Lombardia Cardiac Arrest Registry (Lombardia CARe - NCT03197142) is a multicenter longitudinal prospective Utstein-based registry enrolling all the OHCA cases occurring in the Province of Pavia since January 1, 2015 and in the provinces of Pavia, Lodi, Cremona, and Mantua since January 1, 2019. All the data are collected following Utstein 2014 recommendations (10). Each dataset regarding the pre-hospital treatment of each cardiac arrest that occurred outside of the hospital and for which the Emergency Medical System (EMS) is alerted is automatically captured from the data warehouse of the regional EMS (Agenzia Regionale Emergenza Urgenza – AREU) and filed in the database. For each province one or more EMS investigators are asked to correct and verify the pre-hospital data and one or more clinical investigators for each hospital are in charge of completing data relating to the patient's in-hospital stay. The follow-up is provided both at the outpatient in-hospital visits and by telephone or using data available in the regional health electronic system (Sistema Informativo Socio Sanitario – SISS). The Lombardia CARe Study Management Team is responsible for quality control of all the data entered in the database. Lombardia CARe was approved by the Ethical Committee of the Fondazione IRCSS Policlinico San Matteo (proc. 20140028219) and by all others who were territorially involved. An informed consent form was signed by all the patients discharged alive.

### EMS Organization and Setting

The total area covered by the Lombardia CARe registry is 7,863 km^2^ divided into the four provinces (Pavia 2,969 km^2^; Lodi 783 km^2^; Cremona 1,770 km^2^; Mantua 2,341 km^2^). Each province has several rural regions and a few urban areas for a total population of 1,547,333 inhabitants (Pavia 545,888; Lodi 230,198; Cremona 358,955; Mantua 412,292) as of January 1, 2020. The EMS dispatch center is unique for the four provinces and coordinates 45 ambulances staffed with basic life support and defibrillation (BLS-D)-trained personnel, and 21 advanced life support (ALS)-trained staffed vehicles (a physician and a specialized nurse or a specialized nurse only). The specialized nurse, if alone in the ALS-staffed vehicle, applies the same ALS protocol, using supraglottic devices instead of tracheal intubation. Five helicopters with a physician and a specialized nurse on board also serve the entire region of Lombardy and another three can intervene from other neighboring regions. In the case of suspected OHCA, the EMS dispatcher activates one to three emergency vehicles (which may include a helicopter) with at least one physician and assists the calling bystander during chest compressions (telephone CPR). The decisions about the attempt and the duration of resuscitation are left to the physician whilst BLS-D-trained personnel are instructed to start resuscitation unless clear signs of death are present (rigor mortis, hypostasis, and injuries not compatible with life).

### Utstein Subgroups

We divided our population according to the Utstein 2014 recommendations (10). “All-EMS treated” included all the OHCA patients in whom CPR was started by EMS and it was recommended for system effectiveness comparisons. For the computation of the other three subgroups, the OHCA patients in whom OHCA was witnessed by EMS were excluded. The three subgroups were composed as follow: “Shockable bystander witnessed” included all the OHCA patients witnessed by a bystander with a first shockable rhythm (which measures system efficacy – also called “Utstein comparator group”). “Shockable bystander CPR” included all the OHCA patients with a first shockable rhythm and in whom CPR was started by a bystander. “Non-shockable bystander witnessed” included all the OHCA patients witnessed by a bystander and with a non-shockable first rhythm.

### Data Management

Study data are collected and managed using REDCap (Research Electronic Data Capture) electronic data capture tools hosted at Fondazione IRCCS Policlinico San Matteo (17, 18). REDCap is a secure, web-based software platform designed to support data capture for research studies, providing (1) an intuitive interface for validated data capture; (2) audit trails for tracking data manipulation and export procedures; (3) automated export procedures for seamless data downloads to common statistical packages; and (4) procedures for data integration and interoperability with external sources.

### Statistical Analysis

Statistical analyses were performed by using the SPSS 25.0 analysis program (SPSS Inc, Chicago, Illinois) and the MedCalc Version 19.6 (MedCalc Software bvba). The categorical variables were compared with the Chi-square test and expressed as numbers and percentages. Continuous variables were tested for normal distribution with the D'Agostino-Pearson test. Normally distributed continuous variables were compared by the Student's *t*-test and expressed as mean value ± standard deviation; variables with non-normal distribution were compared by the non-parametric Mann-Whitney test and presented as median and interquartile range [IQR]. Kruskal-Wallis test was used for the comparison of non-normally distributed continuous variables between independent subgroups.

We performed univariable and multivariable Cox proportional regression analyses to identify predictors of long-term survival according to the literature data (19, 20), and we tested linear assumptions. The outcome was calculated considering the last follow-up available for each patient. We used the Kaplan-Meier method with log-rank test to compare survival curves of the different Utstein groups. Age-specific death rates, retrieved from the Istituto Nazionale di Statistica (ISTAT) (http://dati.istat.it/), were used to compute the standardized mortality ratios (SMR) and its 95%CI for each year of follow-up (21). *P*-values < 0.05 were considered statistically significant.

## Results

### Patients' Characteristics

The total number of confirmed OHCA cases that occurred during the study period was 4,924 (782 in 2015, 719 in 2016, 745 in 2017, 756 in 2018, and 1922 in 2019), and resuscitation was attempted in 3,235 of them (490 in 2015, 441 in 2016, 472 in 2017, 506 in 2018, and 1326 in 2019). The majority of patients were men (58.9%) with a median age of 78 [65–85] years, and the median EMS arrival time was 11 (8–14) min. As expected, the majority of OHCA cases occurred at home (91.6%) and were of medical etiology (78.5%). The events were witnessed in 55% of the cases and CPR was started by bystanders in 43.1%. A total of 16.7% of patients were transported to the hospital after the return of spontaneous circulation (ROSC), whilst 14.1% were transported during CPR. The characteristics of all the EMS-treated patients and of the patients divided in the Utstein subgroups are presented in Table 1 and in Supplementary Table 1 concerning respectively the whole population and the patients discharged alive.

**Table 1:** Characteristics of the patients in whom CPR was started by EMS and of the patients divided in the Utstein categories.

**Variable**	**All EMS-treated** **(*n* = 3,235)**	**Shockable bystander witnessed** **(*n* = 383)**	**Shockable bystander CPR (*n* = 333)**	**Non-shockable witnessed (*n* = 1,393)**	***p***
**Men**, ***n*** **(%)**	1904 (58.9)	301 (78.6)	263 (79)	791 (56.8)	<0.001
**Age, years [IQR]**	78 [65–85]	67 [57–78]	66 [57–77]	80 [68–86]	<0.001
**EMS arrival time, mins [IQR]**	11 [8,–14]	9 [7–13]	10 [8–13]	11 [9–15]	<0.001
**Etiology of arrest**, ***n*** **(%)**					<0.001
Medical	2964 (91.6)	375 (97.9)	324 (97.3)	1242 (89.2)	
Trauma	156 (4.8)	4 (1)	4 (1.2)	87 (6.2)	
Drowning	6 (0.2)	0 (0)	0 (0)	2 (0.1)	
Overdose	13 (0.4)	1 (0.3)	1 (0.3)	5 (0.4)	
Electrocution	2 (0.1)	1 (0.3)	1 (0.3)	1 (0.1)	
Asphyxial (external causes)	75 (2.3)	1 (0.3)	2 (0.6)	47 (3.4)	
Unknown	19 (0.6)	1 (0.3)	1 (0.3)	9 (0.6)	
**OHCA location**, ***n*** **(%)**					<0.001
Home	2538 (78.5)	268 (70)	235 (70.6)	1074 (77.1)	
Nursing residence	289 (8.9)	12 (3.1)	9 (2.7)	162 (11.6)	
Workplace	35 (1.1)	13 (3.4)	11 (3.3)	9 (0.6)	
Street	254 (7.9)	48 (12.5)	42 (12.6)	116 (8.3)	
Public building	75 (2.3)	28 (7.3)	24 (7.2)	22 (1.6)	
Sport	20 (0.6)	11 (2.9)	11 (3.3)	4 (0.3)	
Other	24 (0.7)	3 (0.8)	1 (0.3)	6 (0.4)	
**Witnessed status**, ***n*** **(%)**					
Unwitnessed	953 (29.5)	–	40 (12)	–	
Bystander witnessed	1780 (55)	–	291 (87.4)	–	
Witnessed by EMS	487 (15.1)	–	–	–	
Unknown	15 (0.5)	–	2 (0.6)	–	
**Bystander CPR**, ***n*** **(%)** ^†^	1184 (43.1)	291 (76)	–	582 (41.8)	
**Presenting rhythm**, ***n*** **(%)**			–		
Shockable	551 (17)	–	–	–	
Not shockable	2668 (82.5)	–	–	–	
Unknown	16 (0.5)	–	–	–	
**Mechanical CPR**, ***n*** **(%)**	449 (13.9)	144 (37.6)	120 (36)	150 (10.8)	<0.001
**Adrenaline, mg [IQR]**	1 [0–4]	3 [1–5]	3 [1–5]	1 [0–4]	<0.001
**Amiodarone administered**, ***n*** **(%)**	275 (8.5)	155 (40.5)	140 (42)	56 (4)	<0.001
**Outcome**, ***n*** **(%)**					<0.001
*Death in the field*	2240 (69.2)	56 (17.7)	48 (17.4)	566 (65.6)	
*Transported with ongoing CPR*	456 (14.1)	93 (29.3)	78 (28.3)	144 (16.7)	
*Transported with ROSC*	539 (16.7)	168 (53)	150 (54.3)	153 (17.7)	

*P-values refer to comparison among “shockable bystander witnessed,” “shockable bystander CPR,” and “non-shockable witnessed” groups using the Kruskal-Wallis test*.

*EMS, emergency medical service; OHCA, out-of-hospital cardiac arrest; CPR, cardio-pulmonary resuscitation; ACLS, advanced cardiac life support (i.e., endotracheal intubation, administration of drugs, mechanical CPR); ROSC, return of spontaneous circulation*.

† *Excluding those witnessed by EMS*.

### Long-Term Survival

**Figure 1:**
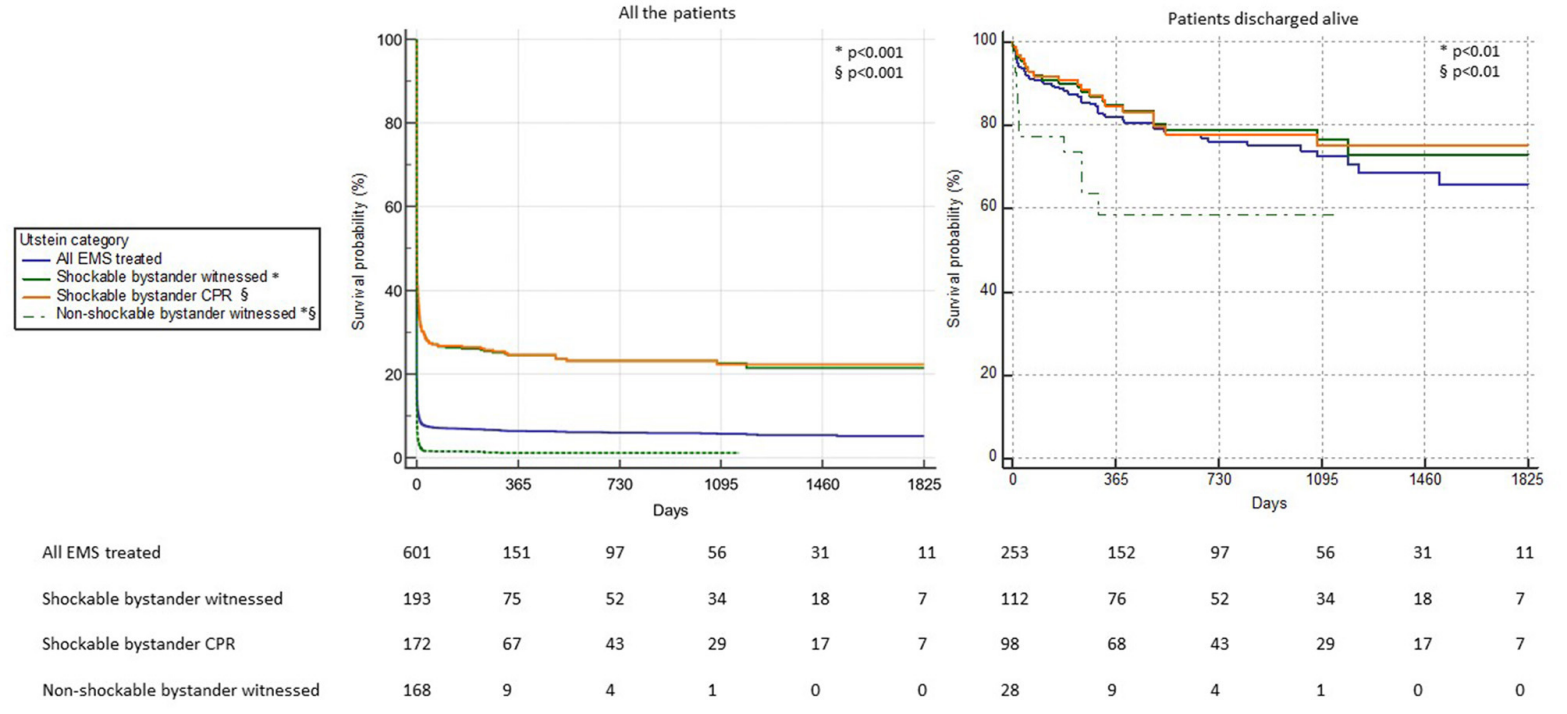
Kaplan-Meier survival curves of the OHCA patients divided into Utstein categories considering all the patients **(Left)** and only the patients discharged alive from the hospital **(Right)**. The statistical comparison between the curves “shockable bystander witnessed” vs. “non-shockable bystander witnessed” (*) and “shockable bystander CPR” vs. “non-shockable bystander witnessed” (§) is reported.

The Kaplan-Maier curves for survival in the different Utstein categories are presented in Figure 1 and show a decreasing survival rate since the beginning and along the entire follow-up in all the Utstein categories. Focusing on patients discharged alive, the survival rate of the “all-EMS treated” group was 82, 76.2, 72.7, 68.7, and 65.9% at the end of the first up to fifth year after the event, respectively. Moving to the other Utstein categories, the Utstein comparator group (“shockable bystander witnessed”) and the “shockable bystander CPR” group behaved in the same fashion as the survival rate dropped from 84.8% at 1 year to 73.1% at 5 years and from 84.8 to 75.3%, respectively. Conversely, the “non-shockable bystander witnessed” group showed a considerable decrease of survival in the very first year of follow-up with a survival rate of 58.3%. The two categories of shockable OHCA (“shockable bystander witnessed” and “shockable bystander CPR”) showed a significantly better survival as compared to “non-shockable bystander witnessed” both when considering the survival from the event and the survival after hospital discharge (*p* < 0.001 for both comparisons, Figure 1).

**Figure 2:**
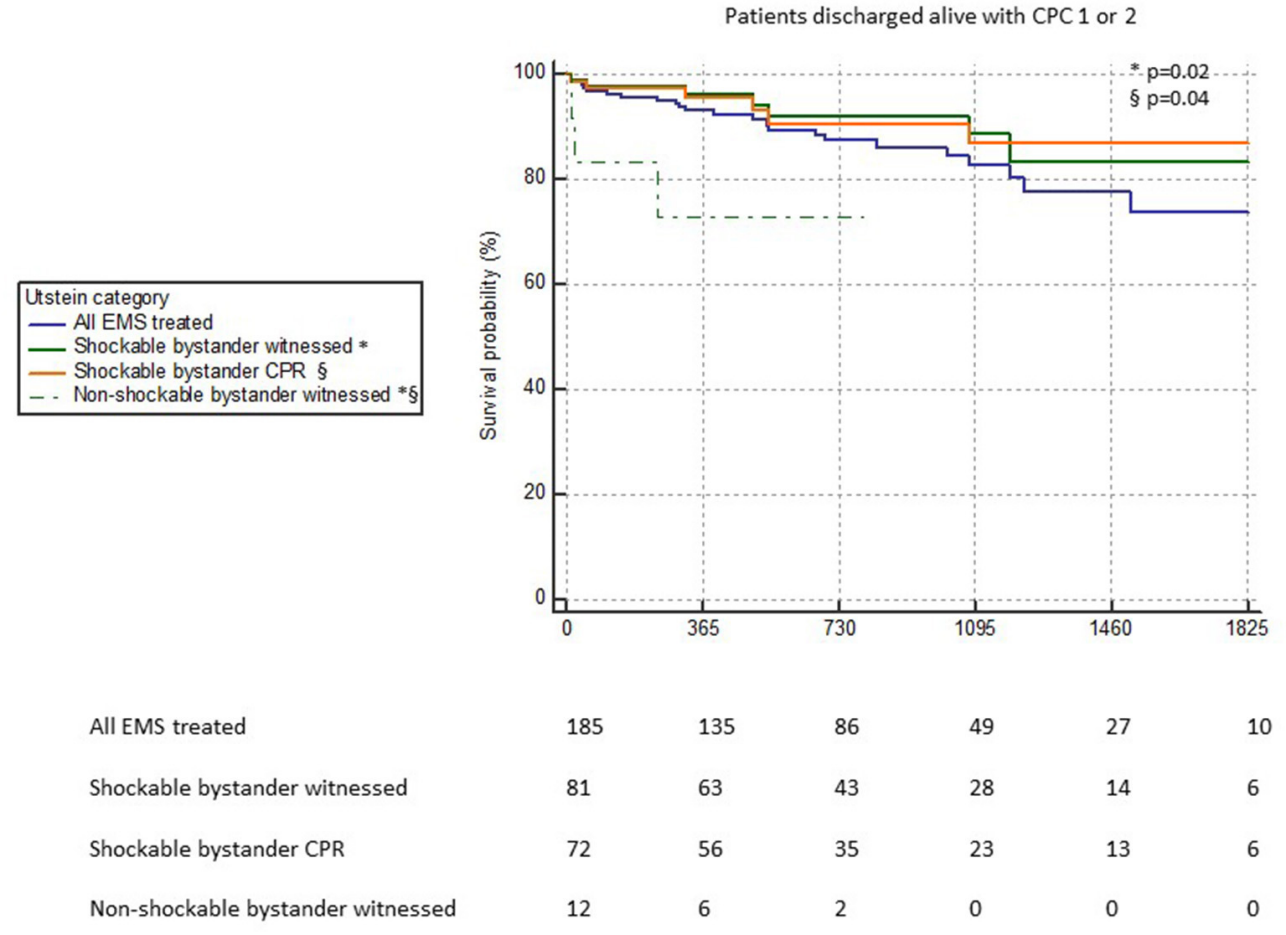
Kaplan-Meier survival curves of the patients discharged alive with good neurological outcome (CPC ≤ 2) divided into Utstein categories. The statistical comparison between the curves “shockable bystander witnessed” vs. “non-shockable bystander witnessed” (*) and “shockable bystander CPR” vs. “non-shockable bystander witnessed” (§) is reported.

A decrease in survival, although milder, was observed in patients discharged alive with good neurological outcome (CPC 1 or 2) as well (Figure 2). The survival rate decreased in 5 years from 93 to 73.7% in the “all-EMS treated” group, from 96 to 83.1% in the Utstein comparator group, and from 95.5 to 86.7% in the “shockable bystander CPR” group. Also in this setting of patients, the “non-shockable bystander witnessed” group showed a decrease of survival along the first year of follow-up reaching 72.9%. Also in this case, there was a statistical significant difference between the curves of “shockable bystander witnessed” vs. “non-shockable bystander witnessed” (*p* = 0.02) and “shockable bystander CPR” vs. “non-shockable bystander witnessed” (*p* = 0.04).

Moreover, a statistical significant difference was highlighted considering the Kaplan-Meier survival curves of the OHCA patients divided according to the presenting rhythm (shockable vs. not shockable; Supplementary Figure 1).

The survival with good neurological outcome (CPC≤2) rate of all the patients treated by EMS and of different Utstein categories is presented in Supplementary Table 2.

### Standardized Mortality Ratios (SMRs)

**Figure 3:**
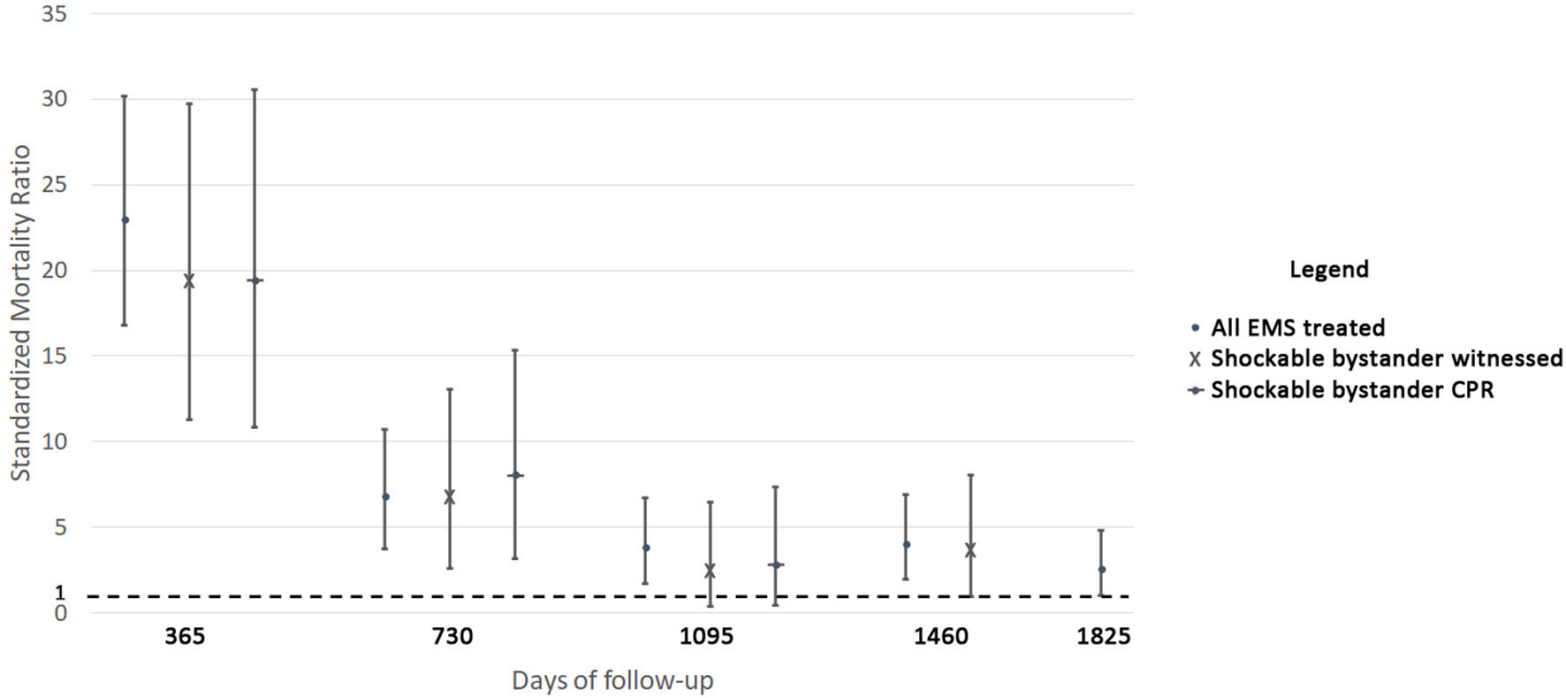
Standardized mortality ratios (SMRs) of the patients discharged alive from the hospital according to Utstein categories.

Yearly SMRs during the follow-up years of the OHCA patients who were alive at hospital discharge are presented in Figure 3. The risk of death of the “all-EMS treated” group was higher than the general population for every year of follow-up: 23 (95%CI 16.8 to 30.2) for the first one, 6.8 (95%CI, 3.8 to 10.7) for the second, 3.8 (95%CI, 1.7 to 6.7) for the third, 4.05 (95%CI, 1.9 to 6.9) for the fourth, and 2.6 (95%CI, 1.03 to 4.8) for the fifth. For the “shockable bystander witnessed” and the “shockable bystander CPR” categories, the risk of death exceeded the general population in the first (19.4, 95%CI 11.3 to 29.8 and 19.4, 95%CI, 10.8 to 30.6, respectively) and in the second (6.8, 95%CI 6.6 to 13 and 8.1, 95%CI, 3.1 to 15.3 respectively) year of follow-up, and became similar from the third one (2.5, 95%CI 0.4 to 6.5 and 2.8, 95%CI, 0.4 to 7.4, respectively).

Analyzing the yearly SMRs of the OHCA patients discharged with a CPC of 1 or 2, similar results were observed for the “all-EMS treated” group, which had a higher risk of death compared with the general population for the first (8.9, 95%CI 5.3 to 13.6), the second (6.6, 95%CI 3.6 to 10.4), the third (5, 95%CI 2.5 to 8.3), and the fourth (9.2, 95%CI 5.8 to 13.4) year of follow-up (Supplementary Figure 2). On the contrary, the risk of death was higher than the general population for all the years of follow-up except for the third one (5.1, 95%CI 1.5 to 10.9; 4.8, 95%CI 1.4 to 10.2; 3.5, 95%CI 0.8 to 8; 5.7, 95%CI 2.1 to 11) for the “shockable bystander witnessed” group, and for all the 3 years of follow-up for the “shockable bystander CPR” group (5.7, 95%CI 1.6 to 12.4; 5.9, 95%CI 1.8 to 12.2; 4.1, 95%CI 1.01 to 9.4).

### Predictors of Long-Term Survival

The univariable Cox regression analysis highlighted that gender, age, sport and public building location, shockable presenting rhythm, witnessed OHCA with bystander CPR, EMS-witnessed OHCA, and EMS arrival time were predictors of long-term mortality (Table 2). However, in multivariable analysis, only age (HR 1.01, 95%CI, 1.0 to 1.01), asphyxial etiology (HR 0.7, 95%CI 0.55 to 0.91), public building location (HR 0.73, 95%CI 0.56 to 0.96), shockable presenting rhythm (HR 0.49, 95%CI 0.43 to 0.55), and EMS-witnessed OHCA (HR 0.76, 95%CI 0.67 to 0.87) were independent predictors of long-term mortality.

**Table 2:** Cox regression analyses of long-term mortality.

	**Unadjusted analysis**			**Adjusted analysis**		
**Survival at discharge**	**HR**	**95% CI**	**P value**	**HR**	**95% CI**	**P value**
**Gender**						
F	Ref			Ref		
M	0.91	0.85 to 0.98	0.012	1.04	0.97 to 1.13	0.28
Age	1.01	1.01 to 1.02	<0.001	1.01	1.00 to 1.01	<0.001
**Etiology**						
Medical	Ref			Ref		
Trauma	1.06	0.90 to 1.25	0.50	1.10	0.90 to 1.33	0.36
Drowning	0.68	0.28 to 1.63	0.38	0.92	0.34 to 2.48	0.87
Overdose	0.90	0.51 to 1.59	0.73	0.87	0.47 to 1.64	0.68
Electrocution	1.23	0.31 to 4.90	0.77	1.57	0.39 to 6.31	0.53
Asphyxial (by external cause)	0.81	0.63 to 1.03	0.08	0.70	0.55 to 0.91	<0.01
**Location**						
Home	Ref			Ref		
Work/office	0.72	0.50 to 1.04	0.08	0.87	0.60 to 1.27	0.47
Sport facilities	0.51	0.30 to 0.88	0.02	0.68	0.39 to 1.17	0.17
Street	0.87	0.76 to 1.00	0.05	0.95	0.81 to 1.12	0.56
Public building	0.59	0.45 to 0.76	<0.001	0.73	0.56 to 0.96	0.02
Long-term care	1.13	0.99 to 1.27	0.06	1.05	0.92 to 1.19	0.48
**Shockable rhythm**						
No	Ref			Ref		
Yes	0.45	0.40 to 0.51	<0.001	0.49	0.43 to 0.55	<0.001
**Witness status and CPR**						
No witnessed, no CPR	Ref			Ref		
No witnessed, yes CPR	0.97	0.84 to 1.11	0.63	1.09	0.95 to 1.26	0.23
Witnessed, no CPR	0.94	0.85 to 1.04	0.25	0.98	0.88 to 1.09	0.69
Witnessed, yes CPR	0.76	0.69 to 0.85	<0.001	0.97	0.87 to 1.09	0.64
EMS witnessed	0.68	0.60 to 0.77	<0.001	0.76	0.67 to 0.87	<0.001
**EMS arrival time**	1.02	1.01 to 1.02	<0.01	1.00	0.99 to 1.01	0.35

## Discussion

### Long-Term Follow-Up and Cardiac Arrest Registries

Long-term survival after out-of-hospital cardiac arrest has not been studied extensively, mainly because of the great challenge of collecting these kinds of data. Our study has two points of strength: it is among the very few covering the long-term survival for all out-of-hospital cardiac arrests and it is the first providing long-term follow-up results for the different Utstein categories. These results highlight the importance of evaluating the long-term follow-up of OHCA patients to better comprehend the long-term issues of survivors. It could also serve as a stimulus to encourage a longer follow-up in the next Utstein style revision.

The latest revision of the Utstein style recommends the collection of survival at hospital discharge or at 30 days after the event, and considers survival at 12 months simply supplemental because of the challenge of such a long-term follow-up (10). This kind of recommendation is reflected in the vast majority of OHCA registries collecting patients worldwide. In Europe, the Danish Cardiac Arrest Registry (22), the Swedish Cardiac Arrest Register (SCAR) (23), and the UK Out-of-hospital Cardiac Arrest Outcome (OHCAO) project (24) focus mainly on 30-day survival as the outcome. The primary outcome of the Pan-Asian Resuscitation Outcomes Study (PAROS) is instead survival to hospital discharge or survival at 30 days for those patients who have not yet been discharged by the 30th day post-arrest (25). The Australian Resuscitation Outcomes Consortium (Aus-ROC) OHCA epidemiological registry (Epistry) considers survival to hospital discharge as the primary outcome, while long-term follow-up data are not collected systematically across all sites so they are not currently included in the registry (26). In the United States of America, the Cardiac Arrest Registry to Enhance Survival (CARES) was unable to provide long-term follow-up, because obtaining written, informed consent from every survivor represents a prohibitive task (27). In this scenario, the Lombardia CARe registry stands out, aiming to provide a follow-up of up to 5 years after the event and representing one of the few prospective OHCA registries with such a long-term follow-up.

### Long-Term Survival After OHCA

Concerning long-term survival issues, the main results of this paper are that survival after OHCA is progressively decreasing in all the Utstein categories and that mortality of OHCA patients discharged alive from the hospital is higher than the general population, not only considering all the OHCA patients, but also considering those with the most favorable OHCA characteristics and those discharged with a good neurological outcome.

Few studies are present in the literature about the outcome of OHCA victims beyond 1 year after the event. A Norwegian study (28) published in 2004 reported 74% survival at 1 year and 41% at 5 years among survivors discharged alive from the hospital. However, since that study was carried out between 1971 and 1992, it is not properly comparable with recent results as the clinical practice and treatment has radically changed. Another study from the 1900's in the U.S. highlighted a better survival in patients aged 65 or less, with a long-term survival similar to the general population, but they included only the patients with first shockable presenting rhythm who received rapid defibrillation (29). Selection was also biased in a recent study from Spain (30), where only patients admitted alive in the Acute Cardiac Care Unit were included. Significant morbidities and mortality were observed in the short- and long-term period in this cohort of patients, identifying a negative neurological outcome at discharge, a non-shockable presenting rhythm, a long collapse to resuscitation time, age, and a low ejection fraction at discharge as predictors of a worse prognosis.

Our study, which includes all the OHCA cases that occurred in four provinces and were filed according to the Utstein style recommendations, should overcome selection bias. This is similar to four studies carried out in Israel (31), Canada (32), and Australia (33–35). Marcus et al. (31) looked for differences in both survival and survival with good neurological outcome between patients aged over and under 80 years. They showed how younger patients had a better outcome, which is consistent with our results, as we also confirmed age as a direct predictor of long-term mortality. Shuvy et al. (32) considered all the OHCA cases that occurred in the Toronto area during a 10-year period reaching results similar to ours. They indeed highlighted that all-cause mortality rates after discharge were 4.3% at 30 days, 12.6% at 1 year, and 20% at 3 years, and that older age was a risk factor of long-term mortality and that shockable presenting rhythm was associated with lower long-term mortality. Andrew et al. (33, 34), in two subsequent papers, analyzed data from the Victorian Ambulance Cardiac Arrest Registry showing that baseline comorbidity may affect long-term mortality of OHCA patients and that the survival decreased after the event from 92.2% at 1 year to 62.3% at 15 years. More interestingly, they proved a standardized mortality ratio (SMR) higher than the general population especially during the first years of follow-up. An SMR higher than the general population was also highlighted in a previous paper focusing on a smaller population from the Stavanger region (36). This evidence was confirmed in our paper, as the SMR was higher, considering the all EMS-treated OHCA, in the first 5 year after the event. However, in our paper we went further, analyzing not only the all EMS-treated OHCAs, but also all the different Utstein subgroups of OHCA patients. We indeed unexpectedly highlighted that the SMR is higher than the general population in those patients with more favorable OHCA characteristics, represented by the Utstein comparator group (shockable bystander witnessed) and the “shockable bystander CPR.” Our results are also in line with another recent Australian paper (35), which showed that patients with non-shockable arrests continued to experience disproportionately higher mortality than patients with an initial shockable arrest in long-term follow-up. Our results, taking into account other variables as suggested by Utstein categories and comparing the mortality risk to the general population, provide new useful information for the long-term management of surviving patients.

This evidence stresses both the importance of a long-term follow-up of OHCA patients to better comprehend the long-term issues of survivors and the utility to use the subgroups of patients suggested by Utstein to also evaluate the long-term outcome. This is also reinforced considering that, in our analysis, we highlighted a similar trend in SMR considering the patients discharged alive with a good neurological outcome (CPC 1 or 2), so excluding those patients with a CPC of 3 or 4 at discharge, which is a recognized predictor of mortality during follow-up (30). The higher mortality risk compared to the general population emphasizes the need both of future research focus on this specific topic to comprehend the reasons for a higher SMR in patients discharged with a good neurological outcome and of strictly monitoring the OHCA patients discharged alive, regardless of the neurological outcome, to prevent possible future complications and early death in the follow-up. The use of the Utstein subgroups even during follow-up would also allow for the comparison of the same type of OHCA patients across different countries, avoiding the selection bias typical of “all EMS-treated patients,” that may result from the different percentage of attempted resuscitation in different countries with very different incidences of EMS-treated OHCA per 100,000 population (3).

Another element worth discussing concerns patients in the Utstein subcategory of “non-shockable bystander witnessed.” In this group of patients, survival impressively decreased along the first 1 year after the event. Such an observation could serve as a guide for an eventual implantation of an internal cardiac defibrillator (ICD), if clinically useful, as secondary prevention in these patients. According to the 2015 ESC Guidelines (37), a reasonable expectation of survival with a good functional status >1 year is required to consider a patient eligible for ICD implantation. Therefore, in light of our results, future studies should focus on the Utstein subgroups to help clinicians in the decision process of implanting a device immediately after the event or after a period of further observation considering the possible presence of various extra-cardiac complications which can negatively affect survival in this specific group of patients.

Our study has limitations. The first limitation is that we were not able to collect the cause of death for all our patients, therefore we used the all-cause mortality. This represents a point of improvement as the cause of death may help to comprehend how to improve the treatment of our patients.

The second limitation is that, in the Utstein subgroups analysis, some patients are included in more than one category, and this prevented us from performing statistical comparisons among the Kaplan-Meier curves of all the patients enrolled and the other subgroups and between the Kaplan-Meier curves of the two subgroups with shockable rhythm. However, this type of bias is implicit in the analysis of the subcategories of patients suggested by Utstein. Moreover, we performed a comparison among the Kaplan-Meier curves of the patients in the two Utstein subcategories with shockable rhythm and the Utstein subcategory with not shockable rhythm, and we performed a comparison between the Kaplan-Meier curves of the patients according only to the presenting rhythm.

The third limitation is that not all of our patients had a 5-year follow-up as we included all the patients enrolled in our registry from 2015 to 2019, with the end of follow-up at June 2020. However, all the patients had at least 6 months of follow-up available, which is longer than the 30-day outcome collected by the majority of OHCA registries.

Another aspect to be considered is that our region was deeply affected by the COVID-19 pandemic since March 2020 (38), overlapping 3 months of our follow-up. Considering the increase in mortality which has been highlighted in our region (39), it could have affected patients' survival.

In conclusion, our study represents the first Utstein-based analysis about long-term follow-up of OHCA survivors. We showed that mortality of OHCA patients discharged alive is higher than the Italian standard population, not only considering the whole population of OHCA patients, but also those with the most favorable OHCA characteristics and with a good neurological outcome. This highlights the importance of a long-term follow-up of OHCA patients to better comprehend the long-term issues of survivors and represents a stimulus to encourage a longer follow-up in the next Utstein style revision.

## Data Availability Statement

The raw data supporting the conclusions of this article will be made available by the authors, without undue reservation.

## Ethics Statement

The studies involving human participants were reviewed and approved by the Ethical Committee of the Fondazione IRCSS Policlinico San Matteo (proc. 20140028219). The patients/participants provided their written informed consent to participate in this study.

## Author Contributions

EB, SC, and SBu: conceptualization, writing - original draft, writing - review & editing, formal analysis methodology, and data curation. RP, SBe, AC, FG, RB, IR, ET, and CF: data curation and investigation. GS, CM, GD, and LO: supervision. SS: conceptualization, formal analysis, writing - original draft, writing - review & editing, methodology, data curation, and supervision. All authors contributed to the article and approved the submitted version.

## Lombardia CARe Researchers

Guido Francesco Villa, Guido Matiz, Maurizio Migliori, Andrea Pagliosa, Fabrizio Canevari, Antonella Brancaglione, Alessandra Palo, Enrico Contri, Vincenza Ronchi, Antonella De Pirro, Simone Molinari, Vito Sgromo, Martina Paglino, Francesco Mojoli, Moreno Curti, Catherine Klersy, Valeria Musella, Livio Carnevale, Arianna Marioni, Giuseppe Bergamini, Francesca Reali, Ugo Rizzi, Daniele Bussi, Simone Ruggeri, Luigi Moschini, Laura Zanotti, Enrico Storti, Pierpaolo Parogni, Fabio Facchin, Giovanni Buetto, Mario Luppi, Dario Franchi, Matteo Caresani, Sabina Campi, Paola Centineo, Roberto De Ponti, Alessandra Russo, Andrea Lorenzo Vecchi, Cecilia Fantoni, Cinzia Franzosi, Claudio Vimercati, Fulvio Giovenzana, Salvatore Ivan Caico, Paola Genoni, Battistina Castiglioni, Marco Botteri, Giovanna Perone, Gianluca Panni, Luca Bettari, Daniele Ghiraldin, Diego Maffeo, Marco Paiella, Umberto Piccolo, Marco Cazzaniga, Ilaria Passarelli.

## Funding

Lombardia CARe is partially funded by the Fondazione Banca del Monte di Lombardia. EB's salary was partially funded by grant 733381 from the European Union Horizon 2020 Research and Innovation Program of ESCAPE-NET.

## Conflict of Interest

The authors declare that the research was conducted in the absence of any commercial or financial relationships that could be construed as a potential conflict of interest.

## Publisher's Note

All claims expressed in this article are solely those of the authors and do not necessarily represent those of their affiliated organizations, or those of the publisher, the editors and the reviewers. Any product that may be evaluated in this article, or claim that may be made by its manufacturer, is not guaranteed or endorsed by the publisher.

## Supplementary Material

Supplementary Figure 1 Kaplan-Meier survival curves of the OHCA patients according to the presenting rhythm (shockable vs. not shockable) considering all the patients (upper left), only the patients discharged alive from the hospital (upper right), and only the patients discharged alive with good neurological outcome (CPC ≤ 2) (lower).

Supplementary Figure 2 Standardized mortality ratios (SMRs) of the patients discharged alive with good neurological outcome (CPC 1 or 2) from the hospital according to Utstein categories.

Supplementary Table 1 Characteristics of the patients in whom CPR was started by EMS and who survived at discharge considering all the patients and Utstein categories.

Supplementary Table 2 Survival with good neurological outcome (CPC ≤ 2) rate of the patients in whom CPR was attempted by EMS considering all the patients and Utstein categories.

Supplementary Table 3 Outcome of the patients in whom CPR was attempted by EMS considering all the patients and Utstein categories (considering survival as an outcome).
