# Supplementary figures and images for: Long-Term Outcome After Out-of-Hospital Cardiac Arrest: An Utstein-Based Analysis
Source: Front Cardiovasc Med. 2021 Dec 15;8:764043. doi: 10.3389/fcvm.2021.764043 (PMC8715950; doi:10.3389/fcvm.2021.764043)

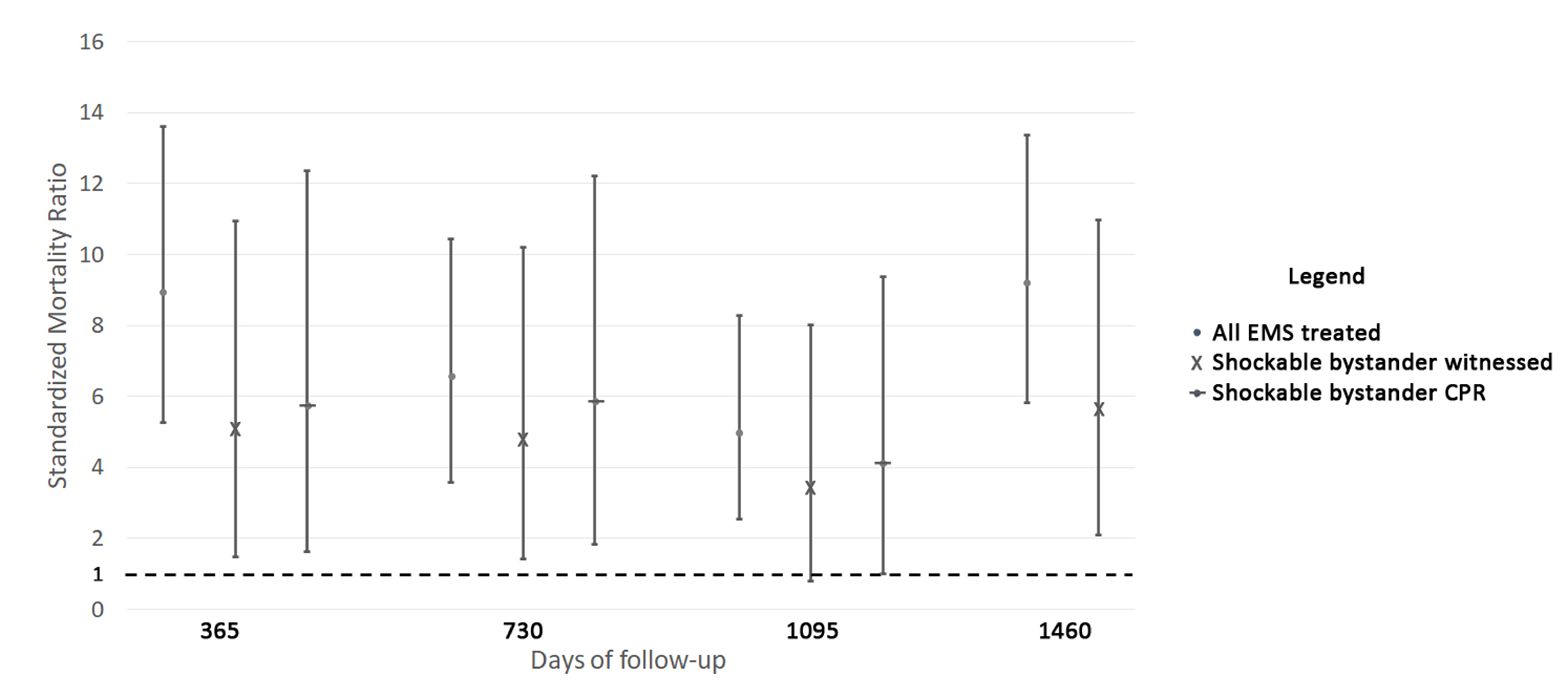

Supplement: Supplementary Figure 1 — Kaplan-Meier survival curves of the OHCA patients according to the presenting rhythm (shockable vs. not shockable) considering all the patients (upper left), only the patients discharged alive from the hospital (upper right), and only the patients discharged alive with good neurological outcome (CPC ≤ 2) (lower). [file Image_1.JPEG]

## Slide 1
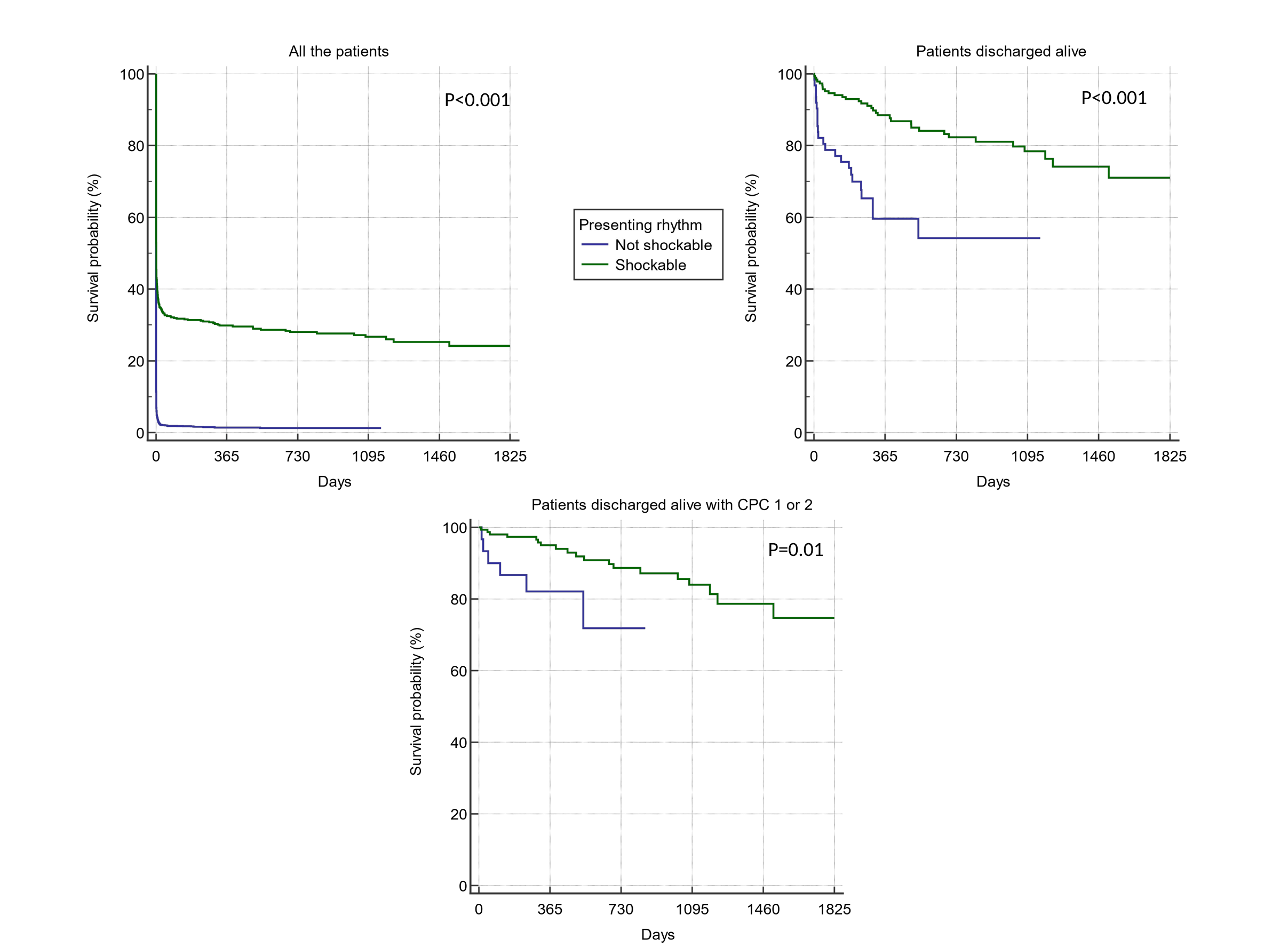

P<0.001
P<0.001
P=0.01

Supplement: Supplementary Figure 2 — Standardized mortality ratios (SMRs) of the patients discharged alive with good neurological outcome (CPC 1 or 2) from the hospital according to Utstein categories. [file Presentation_1.PPTX]
