# Supplementary material for: Long-Term Outcome After Out-of-Hospital Cardiac Arrest: An Utstein-Based Analysis
Source: Front Cardiovasc Med. 2021 Dec 15;8:764043. doi: 10.3389/fcvm.2021.764043 (PMC8715950; doi:10.3389/fcvm.2021.764043)
Supplement: Supplementary Table 1 — Characteristics of the patients in whom CPR was started by EMS and who survived at discharge considering all the patients and Utstein categories. [file Table_1.DOCX]

# **Supplementary Table 1.** Characteristics of the patients in whom a CPR has been started by EMS and who survived at discharge considering all the patients and Utstein categories.

| ***Variable*** | ***All EMS treated***  ***(n=252)*** |  | ***Shockable bystander witnessed***  ***(n=111)*** | ***Shockable bystander CPR (n=97)*** | ***Non-shockable witnessed (n=28)*** | ***p*** |
| --- | --- | --- | --- | --- | --- | --- |
| **Males, n (%)** | 178 (70.6) |  | 83 (74.8) | 75 (77.3) | 14 (50) | 0.013 |
| **Age, years [IQR]** | 64 [55-74] |  | 61 [52-70] | 61 [53-69] | 70 [58-74] | 0.18 |
| **EMS arrival time, mins [IQR]** | 10 [7-13] |  | 9 [6-11] | 9 [7-11] | 9 [7-12] | 0.68 |
| **Etiology of arrest, n (%)** |  |  |  |  |  | <0.001 |
| *Medical* | 231 (91.7) |  | 110 (99.1) | 96 (99) | 19 (67.9) |  |
| *Trauma* | 6 (2.4) |  | 1 (0.9) | 1 (1) | 2 (7.1) |  |
| *Drowning* | 1 (0.4) |  | 0 (0) | 0 (0) | 0 (0) |  |
| *Overdose* | 1 (0.4) |  | 0 (0) | 0 (0) | 1 (3.6) |  |
| *Electrocution* | 0 (0) |  | 0 (0) | 0 (0) | 0 (0) |  |
| *Asphyxial (external causes)* | 13 (5.2) |  | 0 (0) | 0 (0) | 6 (21.4) |  |
| *Unknown* | 0 (0) |  | 0 (0) | 0 (0) | 0 (0) |  |
| **OHCA location, n (%)** |  |  |  |  |  | 0.29 |
| *Home* | 172 (68.3) |  | 63 (56.8) | 54 (55.7) | 21 (75) |  |
| *Nursing residence* | 6 (2.4) |  | 2 (1.8) | 2 (2.1) | 3 (10.7) |  |
| *Workplace* | 8 (3.2) |  | 5 (4.5) | 5 (5.2) | 0 (0) |  |
| *Street* | 31 (12.3) |  | 18 (16.2) | 16 (16.5) | 3 (10.7) |  |
| *Public building* | 21 (8.3) |  | 14 (12.6) | 12 (12.4) | 1 (3.6) |  |
| *Sport* | 7 (2.8) |  | 7 (6.3) | 7 (7.2) | 0 (0) |  |
| *Other* | 7 (2.8) |  | 2 (1.8) | 1 (1) | 0 (0) |  |
| **Witnessed status, n (%)** |  |  |  |  |  |  |
| *Unwitnessed* | 20 (7.9) |  | - | 2 (2.1) | - |  |
| *Bystander witnessed* | 141 (56) |  | - | 95 (97.9) | - |  |
| *Witnessed by EMS* | 91 (36.1) |  | - | - | - |  |
| *Unknown* | 0 (0) |  | - | 0 (0) | - |  |
| **Bystander CPR, n (%)** † |  |  | 95 (85.6) | - | 10 (35.7) |  |
| **Presenting rhythm, n (%)** |  |  |  |  |  |  |
| *Shockable* | 186 (73.8) |  | - | - | - |  |
| *Not shockable* | 63 (25) |  | - | - | - |  |
| *Unknown* | 3 (1.2) |  | - | - | - |  |
| **Mechanical CPR, n (%)** | 44 (17.5) |  | 32 (28.8) | 27 (27.8) | 3 (10.7) | 0.14 |
| **Adrenaline, mg [IQR]** | 0 [0-2] |  | 1 [0-2] | 0.5 [0-2] | 0.5 [0-1.5] | 0.84 |
| **Amiodarone administered, n (%)** | 44 (17.5) |  | 33 (29.7) | 26 (26.8) | 2 (7.1) | 0.049 |
| **Outcome, n (%)** |  |  |  |  |  | 0.7 |
| *Transported with ongoing CPR* | 12 (4.8) |  | 6 (5.4) | 3 (3.1) | 1 (3.6) |  |
| *Transported with ROSC* | 240 (95.2) |  | 105 (94.6) | 94 (96.9) | 27 (96.4) |  |
| **Median follow-up, days [IQR]** | 378 [230-947] |  | 499 [250-1110] | 473 [252-1106] | 243 [21-439] | <0.001 |

EMS: emergency medical service; OHCA: out-of-hospital cardiac arrest; CPR: cardio-pulmonary resuscitation; ACLS: advanced cardiac life support (i.e. endotracheal intubation, administration of drugs, mechanical CPR); ROSC: return of spontaneous circulation.

* Differences are expressed as rate difference or mean difference and 95% confidence interval

† Excluding those witnessed by EMS
