# Supplementary material for: Long-Term Outcome After Out-of-Hospital Cardiac Arrest: An Utstein-Based Analysis
Source: Front Cardiovasc Med. 2021 Dec 15;8:764043. doi: 10.3389/fcvm.2021.764043 (PMC8715950; doi:10.3389/fcvm.2021.764043)
Supplement: Supplementary Table 2 — Survival with good neurological outcome (CPC ≤ 2) rate of the patients in whom CPR was attempted by EMS considering all the patients and Utstein categories. [file Table_2.DOCX]

**Supplementary Table 2**. Survival with good neurological outcome (CPC≤2) rate of the patients in whom a CPR has been attempted by EMS considering all the patients and Utstein categories

|  |  | **Survived event** | **CPC ≤ 2 at discharge** | **CPC ≤ 2 at 30 days** | **CPC ≤ 2 at 6 months** | **CPC ≤ 2 at**  **1 year** | **CPC ≤ 2 at 2 years** | **CPC ≤ 2 at 3 years** | **CPC ≤ 2 at 4 years** | **CPC ≤ 2 at 5 years** |
| --- | --- | --- | --- | --- | --- | --- | --- | --- | --- | --- |
| **EMS witn. included** | **All EMS treated**  **(n=3235)** | 603/3235 (18.6) | 193/3235 (6) | 200/3235 (6.2) | 194/3235 (6) | 152/2612 (5.8) | 96/1665 (5.8) | 55/1135 (4.8) | 30/698 (4.3) | 11/256 (4.3) |
| **EMS witn. Excluded** | **Shockable bystander witnessed (n=383)** | 191/383  (49.9) | 84/383  (21.9) | 86/383  (22.5) | 86/383 (22.5) | 70/299  (23.4) | 49/210  (23.3) | 30/140  (21.4) | 17/88  (19.3) | 7/31  (22.6) |
|  | **Shockable bystander CPR**  **(n=333)** | 166/333  (49.8) | 75/333  (22.5) | 78/333  (23.4) | 77/333  (23.1) | 61/260  (23.5) | 40/180  (22.2) | 25/112  (22.3) | 15/64  (23.4) | 7/23  (30.4) |
|  | **Non-shockable witnessed**  **(n=1393)** | 168/1393  (12.1) | 12/1393  (0.9) | 12/1393  (0.9) | 16/1393  (1.1) | 11/1143  (0.8) | 3/726  (0.4) | 0/511  (0) | 0/299  (0) | 0/102  (0) |
