# Supplementary material for: Long-Term Outcome After Out-of-Hospital Cardiac Arrest: An Utstein-Based Analysis
Source: Front Cardiovasc Med. 2021 Dec 15;8:764043. doi: 10.3389/fcvm.2021.764043 (PMC8715950; doi:10.3389/fcvm.2021.764043)
Supplement: Supplementary Table 3 — Outcome of the patients in whom CPR was attempted by EMS considering all the patients and Utstein categories (considering survival as an outcome). [file Table_3.DOCX]

**Supplementary Table 3**. Outcome of the patients in whom a CPR has been attempted by EMS considering all the patients and Utstein categories (considering survival as outcome)

|  |  | **Survived event** | **Survival at discharge** | **Survival at 30 days** | **Survival at 6 months** | **Survival at 1**  **year** | **Survival at 2 years** | **Survival at 3 years** | **Survival at 4 years** | **Survival at 5 years** |
| --- | --- | --- | --- | --- | --- | --- | --- | --- | --- | --- |
| **EMS witn. included** | **All EMS treated**  **(n=3235)** | 603/3235 (18.6) | 252/3235 (7.8) | 248/3235  (7.7) | 224/3235  (6.9) | 165/2612  (6.3) | 101/1665  (6.1) | 61/1135  (5.4) | 32/698  (4.6) | 12/256  (4.7) |
| **EMS witn. Excluded** | **Shockable bystander witnessed (n=383)** | 191/383  (49.9) | 111/383  (29) | 109/383  (28.5) | 99/383  (25.8) | 77/299  (25.8) | 53/210  (25.2) | 34/140  (24.3) | 19/88  (21.6) | 8/31  (25.8) |
|  | **Shockable bystander CPR**  **(n=333)** | 166/333  (49.8) | 97/333  (29.1) | 95/333  (28.5) | 88/333  (26.4) | 68/260  (26.2) | 44/180  (24.4) | 29/112  (25.9) | 17/64  (26.6) | 8/23  (34.8) |
|  | **Non-shockable witnessed**  **(n=1393)** | 168/1393  (12.1) | 28/1393  (2) | 22/1393  (1.6) | 24/1393  (1.7) | 15/1143  (1.3) | 5/726  (0.7) | 2/511  (0.4) | 0/299  (0) | 0/102  (0) |
